# TMP269, a small molecule inhibitor of class IIa HDAC, suppresses RABV replication *in vitro*

**DOI:** 10.3389/fmicb.2023.1284439

**Published:** 2023-12-01

**Authors:** Juanbin Yin, Shasha Wang, Shanhui Ren, Zhengji Liang, Junwei Ge, Yuefeng Sun, Xiangping Yin, Xiangwei Wang

**Affiliations:** ^1^State Key Laboratory for Animal Disease Control and Prevention, College of Veterinary Medicine, Lanzhou University, Lanzhou Veterinary Research Institute, Chinese Academy of Agricultural Sciences, Lanzhou, China; ^2^Department of Preventive Veterinary Medicine, College of Veterinary Medicine, Northeast Agricultural University, Harbin, Heilongjiang, China

**Keywords:** histone deacetylase inhibitor, TMP269, rabies virus (RABV), antiviral, autophagy

## Abstract

TMP269, a small molecular inhibitor of IIa histone deacetylase, plays a vital role in cancer therapeutic. However, the effect of TMP269 on the regulation of viral replication has not been studied. In the present study, we found that TMP269 treatment significantly inhibited RABV replication at concentrations without significant cytotoxicity in a dose-dependent manner. In addition, TMP269 can reduce the viral titers and protein levels of RABV at an early stage in the viral life cycle. RNA sequencing data revealed that immune-related pathways and autophagy-related genes were significantly downregulated after RABV infection treated with TMP269. Further exploration shows that autophagy enhances RABV replication in HEK-293T cells, while TMP269 can inhibit autophagy to decrease RABV replication. Together, these results provide a novel treatment strategy for rabies.

## Introduction

Rabies is a fatal zoonotic disease caused by rabies virus (RABV), presenting a public health threat to people (Fooks et al., 2017). Every year, approximately 1.2 to 17 million people are bitten by rabid animals, and approximately 60, 000 of them die from rabies (Cleaveland and Hampson, 2017). RABV is an unsegmented single-stranded negative-stranded RNA virus with a full-length genome of about 12 kb, encoding five structural proteins, namely N, P, M, G, and L proteins (Brunker and Mollentze, 2018). Mature RABV particles are composed of two parts: nucleocapsid and envelope. The nucleocapsid is composed of N, P, and L proteins and forms a ribonucleoprotein complex (RNP) with the viral RNA. RNP plays a very important role in viral replication and transcription. The viral envelope is composed of G and M proteins. Among them, the G protein is the only protein that can induce the body to produce neutralizing antibodies. It also plays a decisive role in the cell tropism of the virus and the ability to adsorb cells, making RABV neurotropic (Finke and Conzelmann, 2005; Albertini et al., 2012; Zhang et al., 2013; Davis et al., 2015).

Acetylation and deacetylation, is an important post-translational modification of proteins, and the protein modification level is dynamically regulated by histone acetyltransferases (HAT) and histone deacetylases (HDACs) (Qin et al., 2017). HDACs play a key role in the homeostasis of the acetylation level and the dynamic regulation of HDACs plays an important role in signaling transduction (Suraweera et al., 2018). Many studies have shown that the deacetylation of HDAC is involved in viral replication by regulating the host’s innate immune response (Ellmeier and Seiser, 2018; Mirzaei et al., 2020). HDAC inhibitors (HDACi) were initially considered to be effective anticancer agents (Granieri et al., 2022; Liu et al., 2022) and recently found to be involved in regulating viral replication (Liu G. et al., 2021). In addition, it has been shown to have antiviral effects in animal models of several virus-associated diseases, such as CVB3-induced viral myocarditis (Zhou et al., 2015).

There are 18 family members of HDACs, which can be divided into 4 types according to their different structures (de Ruijter et al., 2003). Class I is composed of HDAC1-3 and HDAC8 (Zupkovitz et al., 2006); Class II consists of HDAC4-7, HDAC9, and HDAC10 (HDAC4, 5, 7, 9 belong to Class IIa, HDAC6, 10 belong to Class IIb); Class III consists of SIRT1-7; and Class IV has only HDAC11 (Zhang et al., 2002). TMP269, a small molecular inhibitor of IIa histone deacetylase, has a protective effect against central nervous system diseases (Su et al., 2020), however, its role in the regulation of viral replication is still unclear.

In this study, we investigated the effect of TMP269 on RABV replication. Here, we found that TMP269 treatment significantly inhibited RABV replication at concentrations without significant cytotoxicity. In addition, TMP269 reduced viral titers and protein levels of RABV at an early stage in the viral life cycle. RNA-sequencing data revealed that autophagy-related genes were significantly downregulated during RABV infection after being treated with TMP269. Further exploration showed that TMP269 can inhibit autophagy to decrease RABV replication. These results may provide information to increase our understanding of RABV pathogenesis and the mechanisms underlying the interaction between RABV infection and host.

## Materials and methods

### Viruses and cell lines

RABV-GFP virus was used for infection. RABV-GFP is an RABV strain of SAD B19 constructed in our laboratory using reverse genetics technology to express the exogenous gene GFP, which is convenient for subsequent experiments and has no effect on virus replication. HEK-293T cells were propagated in Dulbecco’s Modified Eagle Medium (DMEM) supplemented with 10% fetal bovine serum (FBS) (Biological Industries, BioInd, Shanghai).

### CCK-8 assay

CCK-8 assay (Absin Bioscience, Shanghai) was used to assess the cell viability of HEK-293T cells, according to the reagent instructions protocol. HEK-293T cells were treated with DMSO or TMP269 each in 96-well plates for 48 h. Then, 10 μL of CCK-8 solution was added to each well, and the plate was incubated at 37°C for 2 h. At last, the OD value at 450 nm of each well was measured using a spectrophotometer.

### Western blotting

The protein expression level was identified by Western blotting. In brief, cell precipitate was lysed in RIPA lysis buffer with protease inhibitor (Epizyme, Shanghai). Equal amounts of protein samples were separated by sodium dodecyl sulfate-polyacrylamide gel electrophoresis (SDS-PAGE), transferred to polyvinylidene difluoride (PVDF) membranes, blocked with 5% non-fat milk powder for 2 h at room temperature, and incubated with primary antibody overnight at 4°C followed by washing three times with TBST for 10 min. The primary antibodies included rabbit anti-LC3B (at 1:1000 dilution, Abmart, Shanghai), mouse anti-GAPDH (at 1:5000 dilution, Abmart, Shanghai), rabbit anti-ATG5 (at 1:1000 dilution, Cell Signaling Technology, USA), and rabbit anti-M (at 1:1000 dilution, stored in our laboratory). After that, the membranes were incubated with the corresponding secondary antibody at room temperature for 1 h. Finally, after washing thrice again, the membrane was exposed to a chemiluminescence reagent solution kit (Beyotime, Shanghai) using a multi-chemiluminescence image analysis system (Amersham Imager 600, New York, USA).

### TCID_50_ assay

The TCID_50_ assay was used to determine the viral titers. The Vero-E6 cells were suspended in 96-well plates, and the cells were infected when the cell density was greater than 90%. The collected supernatant from the cells of each group was diluted at a series of 10-fold serial dilutions from 10^−1^ to 10^−8^ with serum-free DMEM medium. Each well of 96-well plates was added to 100 μL of the above samples and then incubated at 37°C for 1 h. Then, the inoculum was removed, and the cells were cultured in DMEM supplemented with 1% FBS for 72 h. The positive wells were observed and recorded with a fluorescence microscope. TCID_50_ was calculated using the Reed–Muench method (Reed and Muench, 1937).

### RNA-seq analysis

Total RNA from each sample was extracted using TRIzol Reagent (Invitrogen, CA, USA) following the manufacturer’s instructions. The purity and concentration of the extracted RNA of each sample were calculated using a NanoDrop 2000 spectrophotometer (Thermo Scientific, USA). RNA integrity was assessed on an Agilent 2,100 Bioanalyzer (Agilent Technologies, USA). The RNA sequencing and analysis were conducted by OE Biotech Co., Ltd. (Shanghai, China). The cDNA libraries were sequenced on an Illumina HiSeq X Ten platform. Differentially expressed genes (DEGs) were analyzed by Hierarchical clustering, and GO enrichment and KEGG pathway enrichment analyses of DEGs were used to investigate gene expression profiles.

### Quantitative real-time PCR

Total RNA was extracted using TRIzol reagent (Invitrogen). RNA was used as the template for cDNA synthesis using PrimeScript™ RT Reagent Kit with gDNA Eraser (TAKARA, Beijing). cDNA was then subjected to real-time PCR quantification using SYBR green Premix Ex Taq II (TAKARA, Beijing). The β-Actin gene was used as an internal control. All the experiments were repeated at least three times, and relative mRNA expression levels were calculated using the threshold cycle (2^–△△Ct^) method.

### RNA interference

siRNA was used to knockdown cellular ATG5 expression. The siRNA sequences against ATG5 were as follows: si-ATG5: sense: 5′-GUCCAUCUAAGGAUGCAAUTT-3′; antisense: 5′-AUUGCAUCCUUAGAUGGACTT-3′; and a non-target control siRNA (sense: si-Ctrl: 5′-UUCUCCGAACGUGUCACGUTT-3′; antisense: 5′-ACGUGACACGUUCGGAGAATT-3′) were designed by Sangon Biotech (Shanghai, China) (Ren et al., 2019). These siRNAs were transfected into HEK-293T cells using jetPRIME (Polyplus, Illkirch, France), according to the manufacturer’s instructions.

### Statistical analysis

The data in this study were analyzed by GraphPad Prism (Version 6.01) software. Intragroup or intergroup differences were analyzed by Student’s *t-test*, and the difference was statistically significant at *p < 0.05, **p < 0.01, ***p < 0.001. Data were presented as mean ± standard deviation.

## Results

### TMP269 Inhibits RABV replication at concentrations that show low cytotoxicity

**Figure 1:**
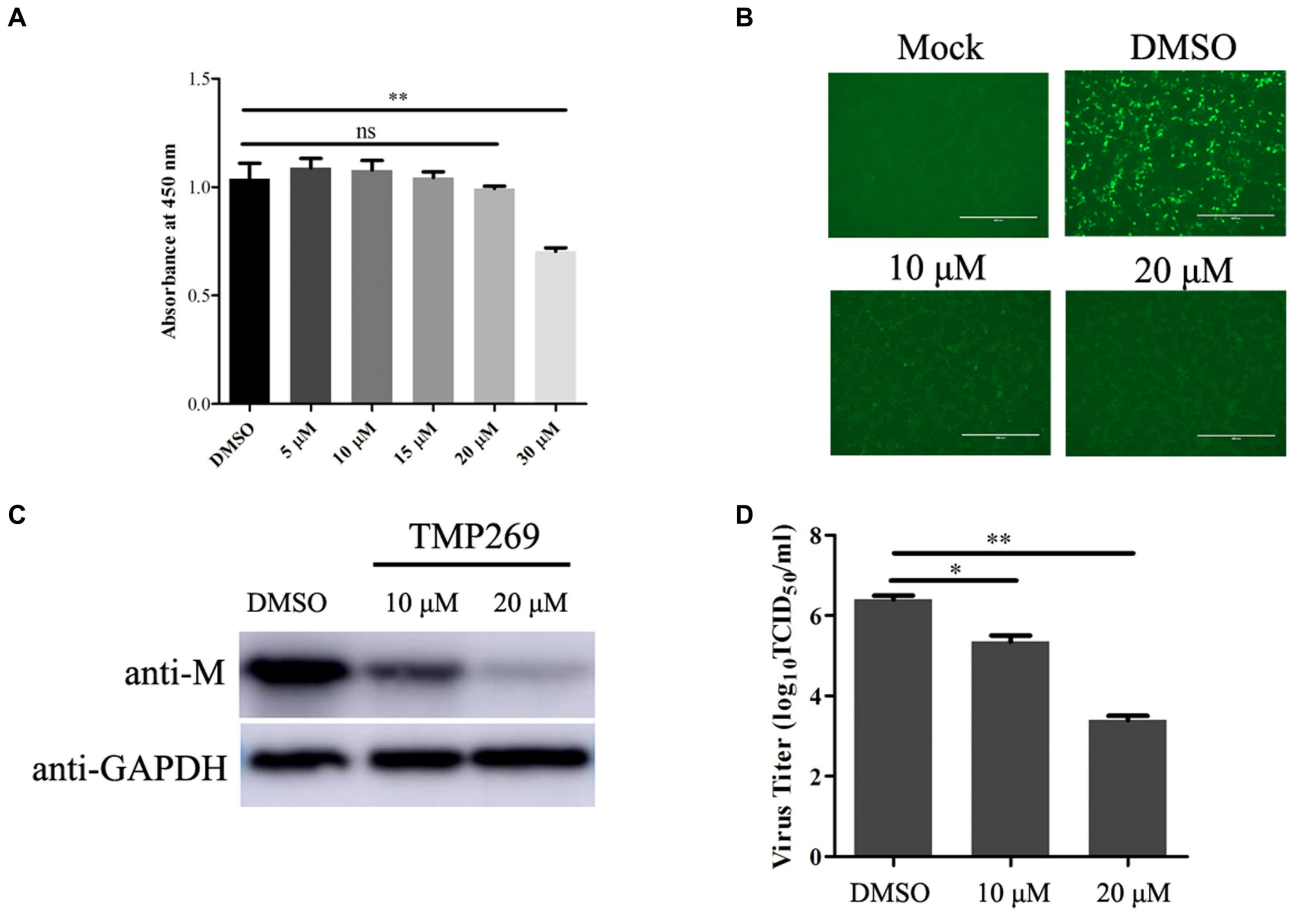
TMP269 inhibits RABV-GFP replication at concentrations that show low cytotoxicity. **(A)** Cytotoxicity of TMP269 determined using CCK-8 assay. HEK-293T was treated with TMP269 for 48 h and then incubated with CCK-8 for 4 h. Relative cell quantities were measured by the value of A450. **(B)** Observation of the green fluorescent expression of RABV-GFP treated with TMP269 (10 or 20 μM) using fluorescence microscopy. **(C)** Western blotting was used to determine the effect of TMP269 on RABV-GFP replication. **(D)** TCID_50_ assay was used to determine the viral titers. *p < 0.05; **p < 0.01.

HDAC inhibitor (HDACi) was recently found to be implicated in the regulation of viral replication; however, the role of TMP269 in RABV replication has not been reported. First, the cell viability was detected using the CCK-8 assay kit after the HEK-293T cells were treated with different doses of TMP269 (10, 20, or 30 μM). The result showed no obvious cell death induced by the indicated concentration except 30 μM (Figure 1A). Next, we detected the effect of TMP269 on RABV replication using a recombinant RABV expressing green fluorescent protein (RABV-GFP). HEK-293T cells were infected with RABV-GFP (0.5 MOI) along with different doses of TMP269 (10 and 20 μM), and the cell samples and supernatant were harvested at 24 hpi. A fluorescence microscope was used to observe the expression of GFP. As shown in Figure 1B, TMP269 can significantly reduce the expression of GFP in a dose-dependent manner. Meanwhile, the expression of M protein (Figure 1C) and the viral titers (Figure 1D) were reduced in a dose-dependent manner. These results suggest that TMP269 can inhibit RABV replication at concentrations that show low cytotoxicity.

### Time course of TMP269-mediated inhibition on RABV replication

**Figure 2:**
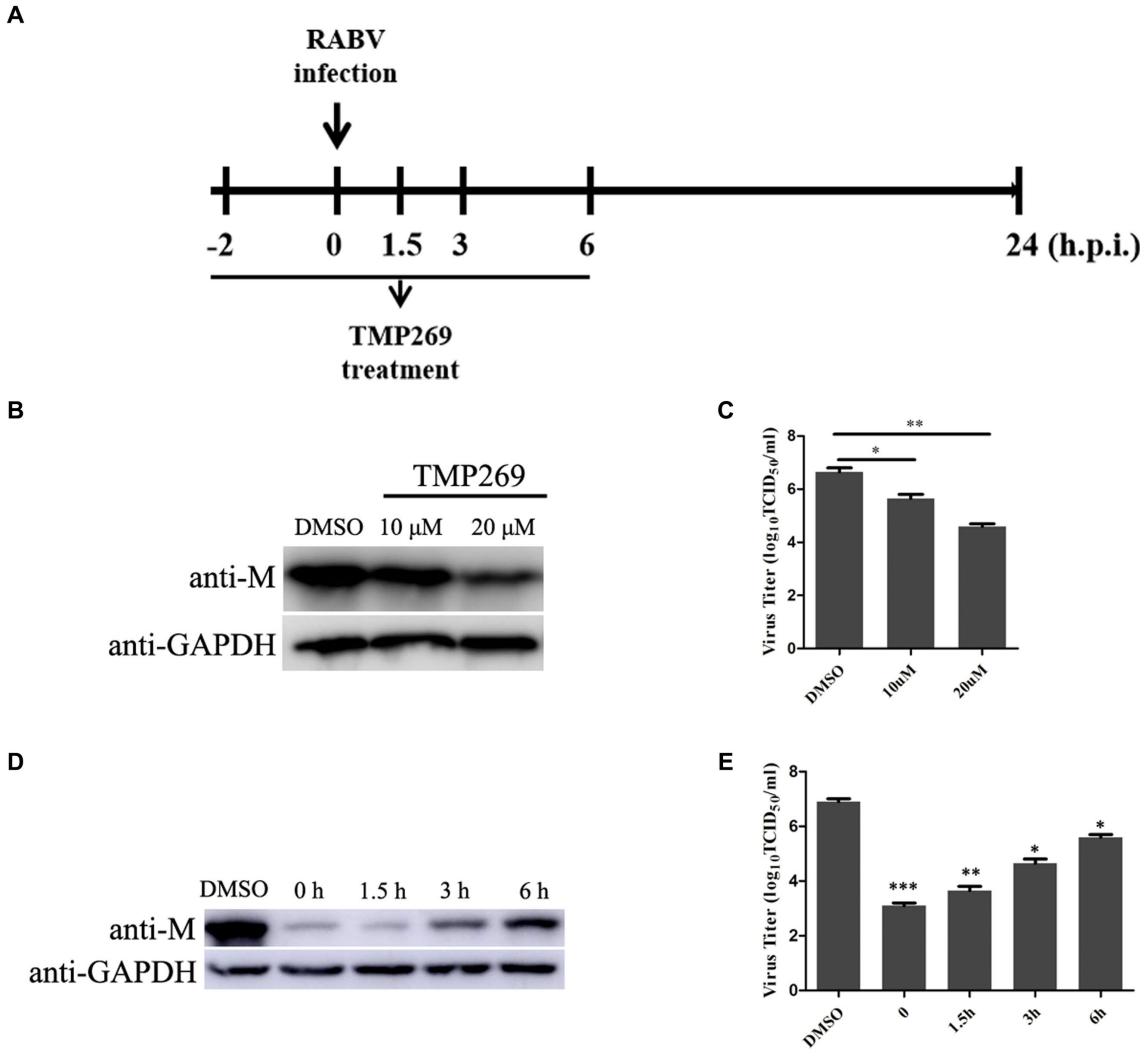
Time course of TMP269-mediated inhibition of RABV-GFP replication. **(A)** Schematic representation of the experimental design. HEK-293T cells were treated with TMP269 (20 μM) at the indicated times. **(B)** The M protein of RABV-GFP expression was analyzed by Western blotting pretreatment with TMP269. **(C)** The viral titer was detected by TCID_50_ assay pretreatment with TMP269. **(D)** The M protein of RABV-GFP expression was analyzed using Western blotting at TMP269 time-of-addition assays. **(E)** The viral titer was detected using the TCID_50_ assay at TMP269 time-of-addition assays. *p < 0.05; **p < 0.01; and ***p < 0.001.

To further illustrate the effect of TMP269 on RABV infection, pretreatment and time-of-addition experiments were performed. Figure 2A shows the methodologies employed for the pretreatment and time-of-addition assays. HEK-293T cells were seeded into 12-cell plates and grown up to 80% confluence. The cells were pretreated to TMP269 (10 and 20 μM) for 2 h, and then, the cells were infected with RABV-GFP (MOI = 0.5) for 24 h without TMP269 incubation, or the cells were infected with RABV-GFP (MOI = 0.5), followed by TMP269 (20 μM) incubated at 0, 1.5, 3, and 6 hpi. At 24 hpi, cell samples and supernatants were collected for Western blotting and TCID_50_ to determine viral titer (Figure 2A). The pretreating assay demonstrated that TMP269 can inhibit RABV-GFP replication both at the protein level (Figure 2B) and the viral titer level (Figure 2C) in a dose-dependent manner. In the time-of-addition assay, marked inhibiting effects on viral replication were observed when TMP269 was added during the immediate early stage of viral replication, and the inhibition effect of TMP269 on RABV replication was decreased along with time (Figures 2D,E). These results indicated that TMP269 mainly affects the early stages of RABV proliferation.

### RNA-seq analysis reveals the difference in the presence or absence of TMP269 during RABV infection

**Figure 3:**
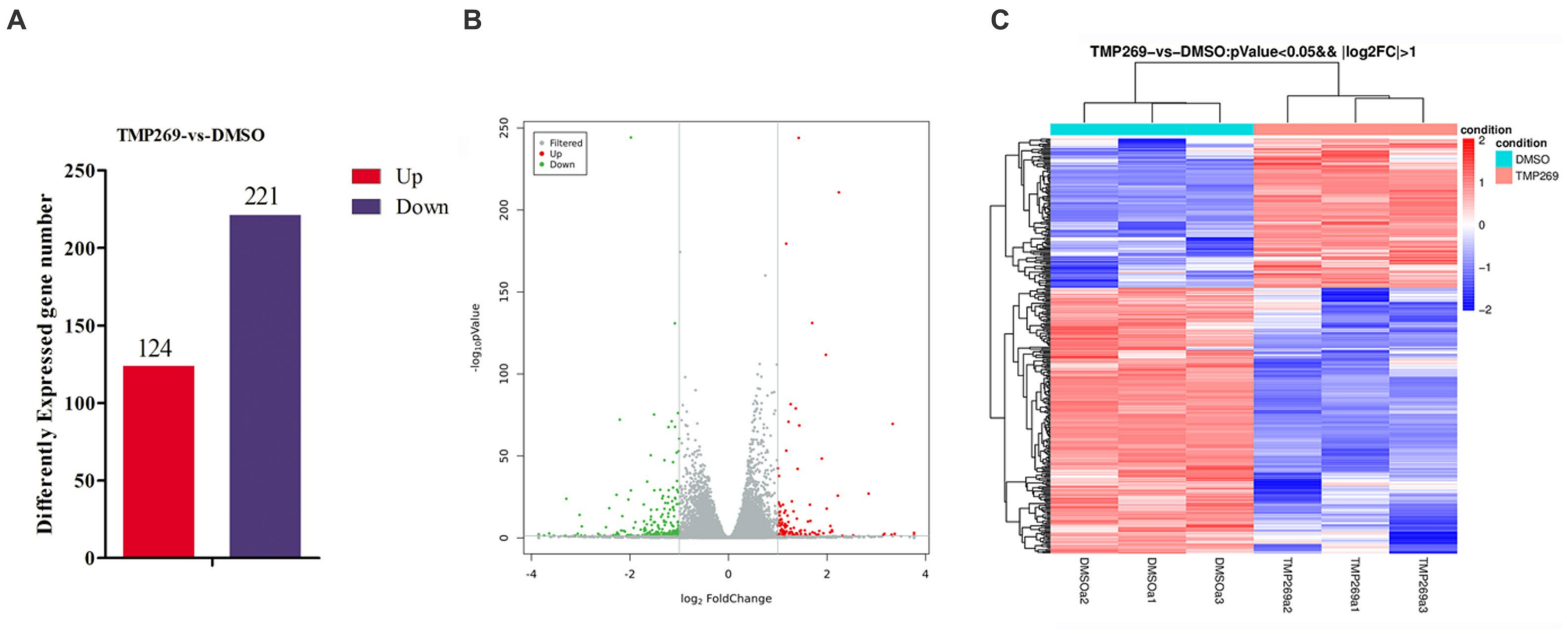
RNA-seq analysis revealed the difference in the presence or absence of TMP269 during RABV-GFP infection. **(A)** The number of differentially expressed genes (DEGs) in the TMP269 group compared with the DMSO group. Red and blue represent the upregulated and downregulated genes, respectively. **(B)** Volcano plot of DEGs. The *x*-axis indicates the base 2 logarithm of fold change; the y-axis indicates the negative logarithm of the value of *p*. **(C)** A heatmap analysis is used to classify gene expression patterns. Red stripes in the figure represent high-expression genes, while blue stripes represent low-expression genes.

To reveal the molecular mechanisms underlying the antiviral effect of TMP269, the HEK-293T cells infected with RABV-GFP for 24 h and treated with DMSO or TMP269 (20 μM) were harvested to carry out RNA sequencing. A *p-*value of <0.05 and |log^2^ fold change|>2 were used as thresholds to identify differentially expressed genes (DEGs). A total of 345 DEGs with 124 promoted DEGs and 221 suppressed DEGs were identified in the TMP269-treated group compared with the DMSO-treated group (Figure 3A). These results were clearly visualized by constructing a volcano plot of the DEGs (Figure 3B) and clustering the samples by differential treatment (Figure 3C). A list of the DEGs is presented in Supplementary Table S1.

### Biological function analysis of DEGs

**Figure 4:**
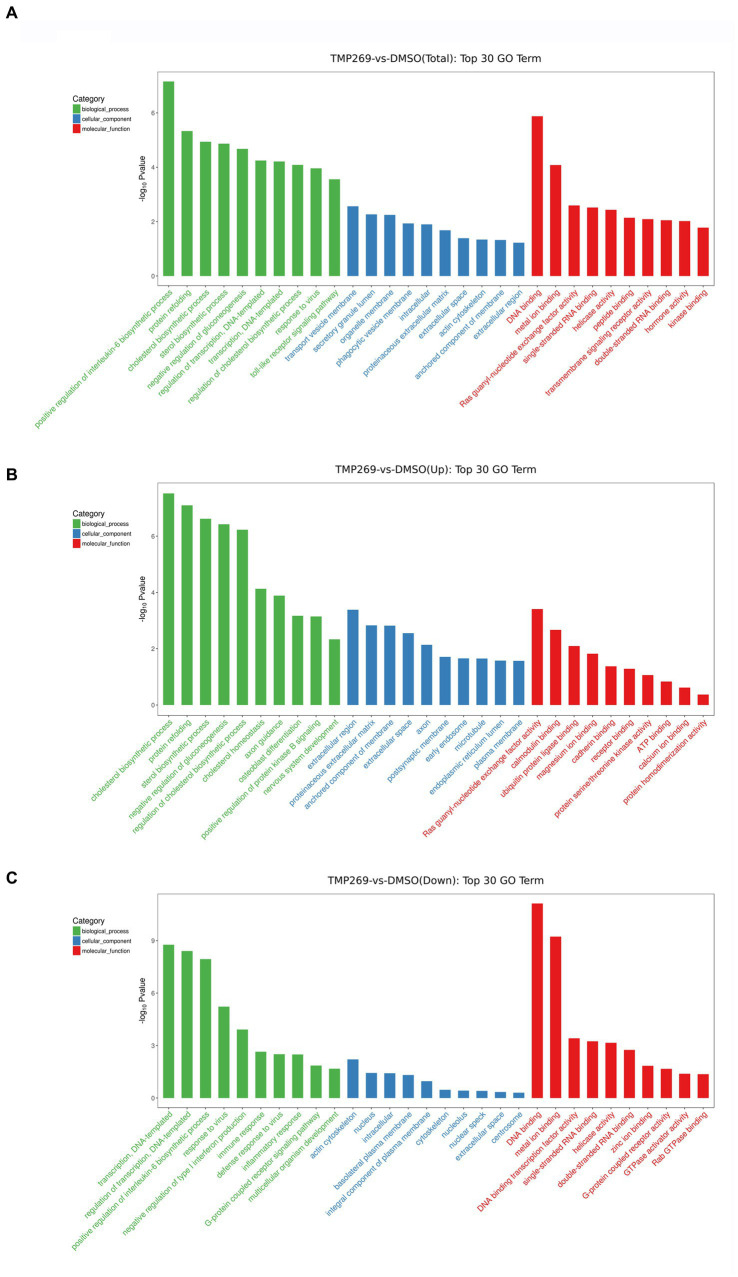
GO categories of DEGs. **(A)** The top 30 most-enriched GO categories of DEGs. **(B)** The top 30 most-enriched GO categories of upregulated DEGs. **(C)** The top 30 most-enriched GO categories of downregulated DEGs. The *x*-axis represents the significantly enriched GO terms and the *y*-axis denotes the negative log value of value of *p*.

**Figure 5:**
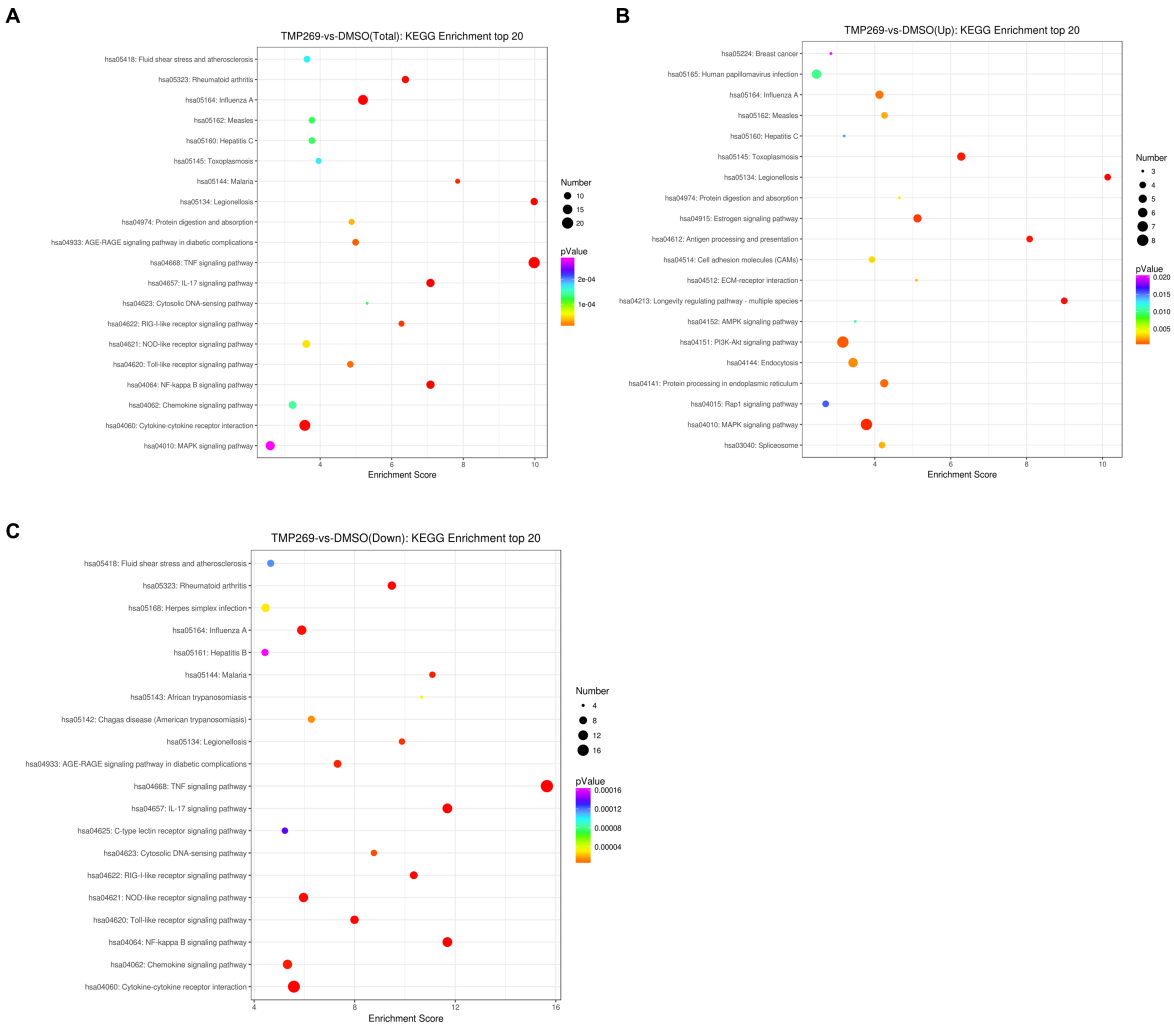
DEG Pathway annotation. **(A)** The top 20 pathways of DEGs. **(B)** The top 20 pathways of the upregulated DEGs. **(C)** The top 20 pathways of the downregulated DEGs. The *x*-axis denotes the pathway enrichment. The *y*-axis represents the names of the significantly enriched pathways.

We then performed GO enrichment and KEGG pathway to analyze the function of these DEGs. Figure 4A shows the GO terms in which DEGs are significantly enriched. According to the GO functions, these DEGs were classified into biological processes, cellular components, and molecular functions. The top 30 biological processes that were significantly enriched in positive regulation of interleukin-6 biosynthetic process (GO:0045410), protein refolding (GO:0042026), regulation of cholesterol biosynthetic process (GO:0045540), and transcription, were DNA-templated (GO:0006351) (Figure 4A). Among these DEGs, the upregulated DEGs were mainly involved in cholesterol biosynthetic process (GO:0006695), protein refolding (GO:0042026), cholesterol homeostasis (GO:0042632), and regulation of cholesterol biosynthetic process (GO:0045540) (Figure 4B), and the downregulated DEGs were mainly involved in transcription, DNA-templated synthesis (GO:0006351), regulation of transcription, DNA-templated synthesis (GO:0006355), positive regulation of interleukin-6 biosynthetic process (GO:0045410), and response to virus (GO:0009615) (Figure 4C). KEGG pathway analysis was used as an additional way to explore the function of the DEGs. The top 20 KEGG pathways were mainly involved in the TNF signaling pathway, Influenza A pathway, Legionellosis pathway, and NF-kappa B signaling pathway (Figure 5A). The significantly enriched KEGG pathways (*p* < 0.05) are presented in Supplementary Table S2. Among these DEGs, the upregulated DEGs were mainly involved in the MAPK signaling pathway, PI3K-Akt signaling pathway, protein processing in endoplasmic reticulum, and endocytosis (Figure 5B and Supplementary Table S3). The downregulated DEGs were mainly involved in the TNF signaling pathway, RIG-I-like receptor signaling pathway, Toll-like receptor signaling pathway, NF-kappa B signaling pathway, and cytokine-cytokine receptor interaction (Figure 5C and Supplementary Table S4).

### TMP269 suppresses RABV-induced immune response

**Figure 6:**
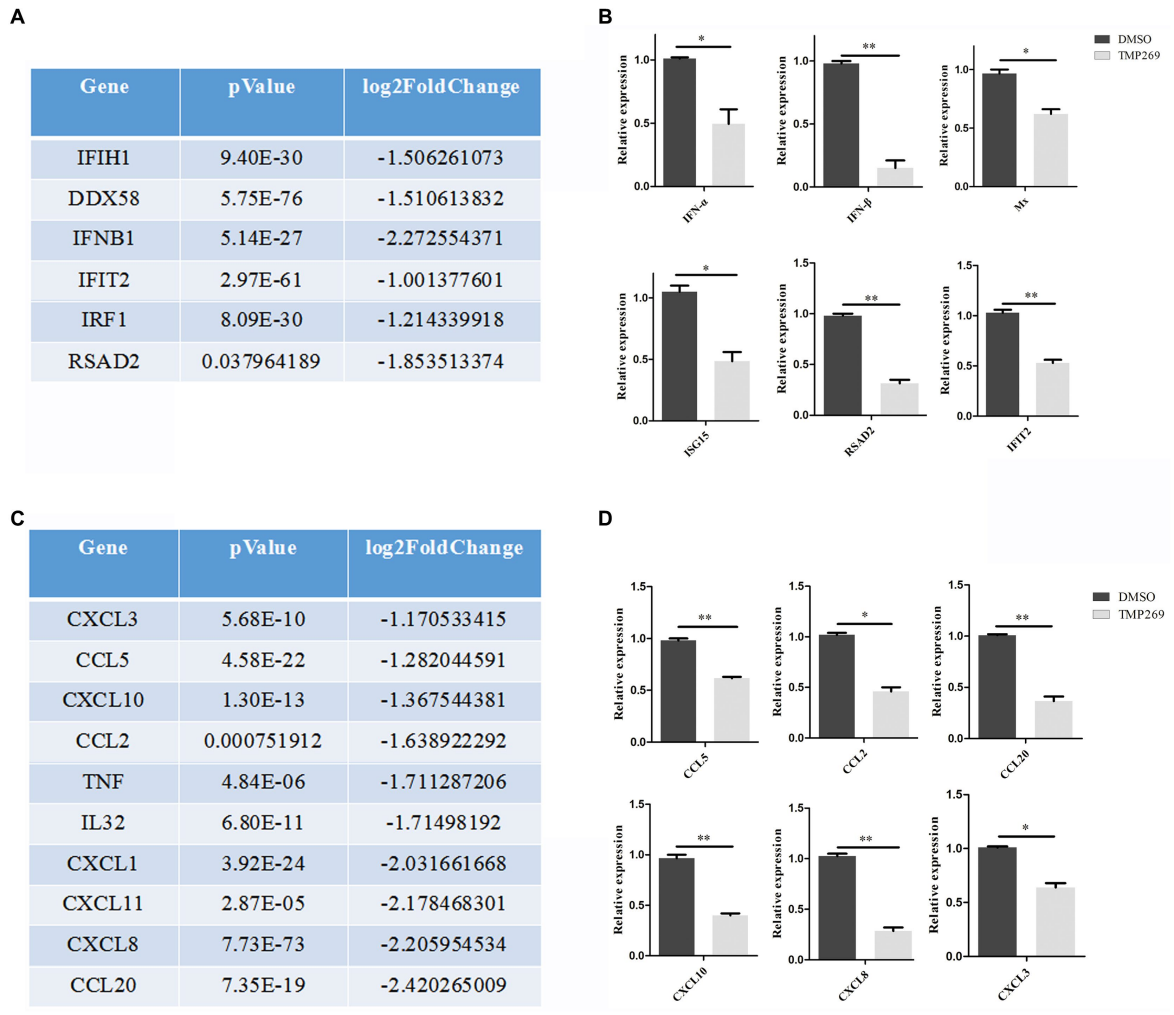
TMP269 suppresses RABV-GFP-induced immune response. **(A)** RNA sequencing analysis focused on molecules in IFN-stimulated genes altered by treatment with TMP269. **(B)** Transcriptions of IFN-α, IFN-β, Mx, ISG15, RSAD2, and IFIT2 genes, measured using qRT-PCR, following treatment with TMP269. **(C)** RNA sequencing analysis focused on molecules and cytokines altered by treatment with TMP269. **(D)** Transcriptions of CCL2, CCL5, CCL20, CXCL3, CXCL8, and CXCL10 genes measured using qRT-PCR, following treatment with TMP269. *p < 0.05; **p < 0.01.

We found that a number of specific pathways were significantly altered in TMP269-treated cells during RABV infection, one of which was associated with innate immune-related pathways (Figure 5A). Data analysis showed that TMP269 reduced the expression of many types of IFN-stimulating genes and cytokines (Figures 6A,C).

IFN-mediated immunity and the inflammatory response play an important role in the defense against virus invasion. In the present study, we measured the expression of interferon (IFN-α and IFN-β) and interferon stimulating genes (Mx, ISG15, RSAD2, and IFIT2) (Figure 6B) and cytokines (CCL2, CCL5, CCL20, CXCL3, CXCL8, and CXCL10) (Figure 6D) and found that transcription levels of these genes were all reduced by TMP269. These data indicate that TMP269 reduces the host’s innate immune response to RABV infection.

### TMP269 inhibits RABV replication by inhibiting autophagy

**Figure 7:**
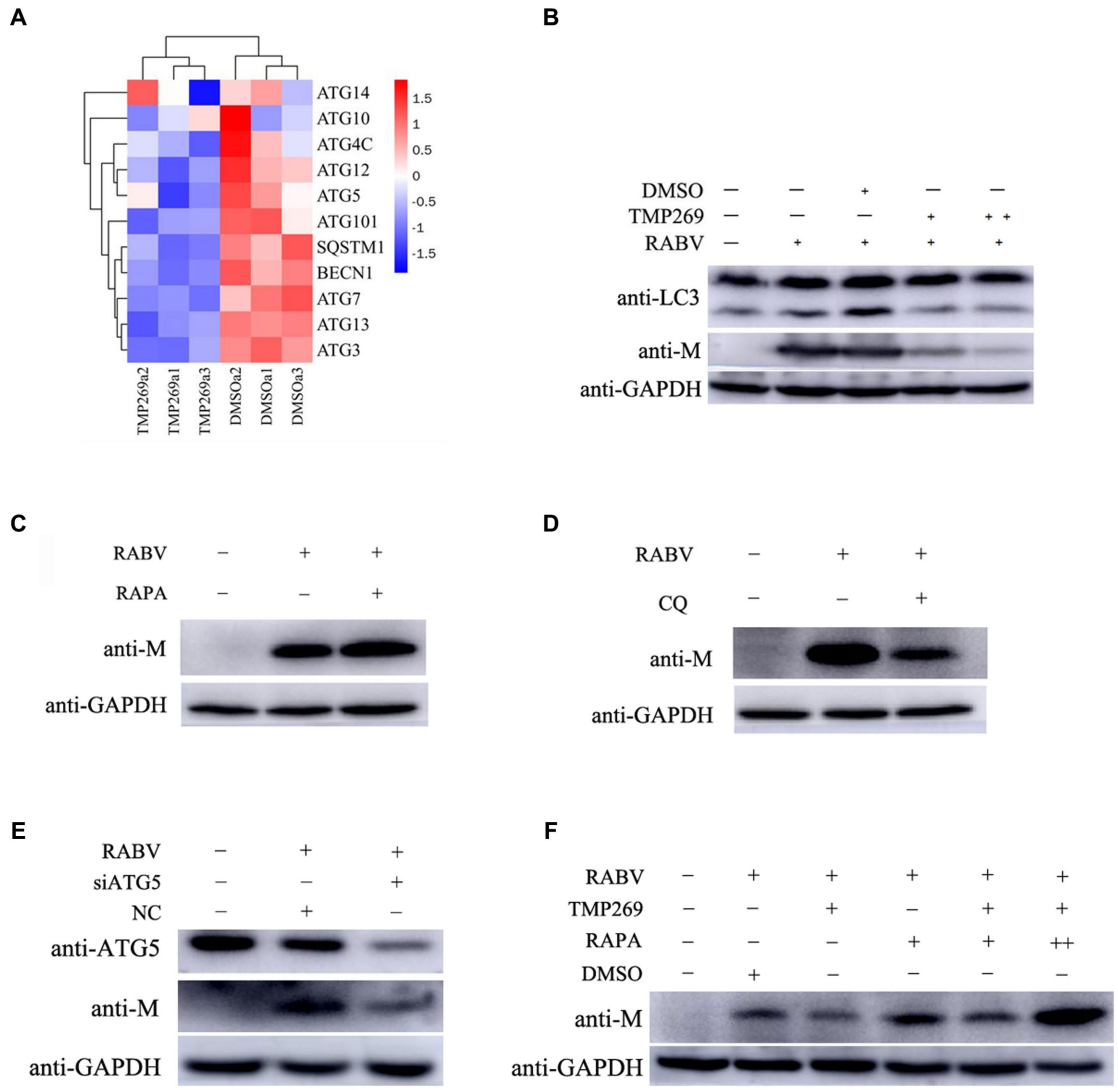
TMP269 inhibits RABV-GFP replication by inhibiting autophagy. **(A)** A heatmap analysis is used to analyze autophagy-related gene expression compared between the TMP269 group and the DMSO group. The red stripes in the figure represent high-expression genes, while the blue stripes represent low-expression genes. **(B)** Western blotting was used to detect the expression of LC3 and M protein in HEK-293T cells infected with RABV-GFP, following treatment with TMP269 or DMSO. **(C)** Western blotting was used to determine the M protein expression of RABV-GFP treated with RAPA. **(D)** Western blotting was used to determine the M protein expression of RABV-GFP treated with CQ. **(E)** HEK-293T cells were transfected with siRNA-ATG5 or scrambled siRNA for 24 h, and then, the cells were infected with RABV-GFP for 24 h. The cell samples were then analyzed using Western blotting with anti-ATG5, anti-M, and anti-GAPDH antibodies. **(F)** Western blotting was used to detect the expression of M protein in HEK-293T cells infected with RABV-GFP, following treatment with TMP269, RAPA, or DMSO.

We used bioinformatics methods to investigate the mechanism of the antiviral effect of TMP269 on RABV by focusing on related pathways or cellular factors. As shown in Figure 7A, the expression of autophagy-related genes in the TMP269 treatment group, such as ATG14, ATG10, ATG4C, ATG12, ATG5, ATG101, SQSTM1, BECN1, ATG7, ATG13, and ATG3, was decreased compared with that in the DMSO treatment group. To investigate whether TMP269 suppresses RABV by inhibiting autophagy, HEK-293T cells were infected with RABV-GFP (0.5 MOI) along with TMP269 (10 and 20 μM); cell samples were harvested at 24 hpi. As shown in Figure 7B, TMP269 treatment can decrease the expression of LC3-II and the replication of RABV-GFP in a dose-dependent manner. Previous studies have shown that autophagy was beneficial to RABV replication in N2a cells and SK cells (Jiaojiao Peng et al., 2016; Liu et al., 2017). In this study, HEK-293T cells pretreated with rapamycin (RAPA) (an autophagy activator) or chloroquine (CQ) (an autophagy inhibitor) can significantly promote (Figure 7C) or inhibit (Figure 7D) the replication of RABV-GFP. In addition, knockdown of the ATG5 by siRNA can significantly reduce the replication of RABV-GFP (Figure 7E), which demonstrated that autophagy was beneficial to RABV replication in the HEK-293T cells. Next, we investigated the inhibition effect of TMP269 on RABV in the presence of RAPA. As shown in Figure 7F, the inhibition effect of TMP269 on RABV was weakened in the presence of RAPA. These data demonstrated that TMP269 inhibited RABV replication by inhibiting the autophagy process.

### TMP269 inhibits RABV replication in N2a cells, Vero cells, and BHK-21 cells

**Figure 8:**
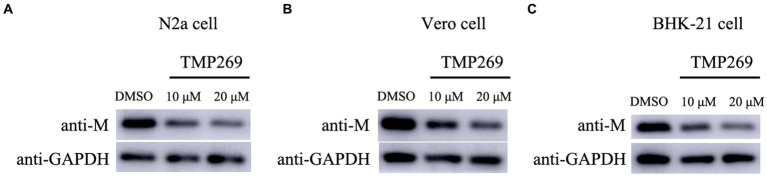
TMP269 inhibits RABV-GFP replication in N2a cells, Vero cells, and BHK-21 cells. Western blotting was used to determine the effect of TMP269 on RABV-GFP replication in N2a cells **(A)**, Vero cells **(B)**, and BHK-21 cells **(C)**.

To further detect the antiviral effect of TMP269 on RABV replication, N2a cells, Vero cells, and BHK-21 cells were infected with RABV-GFP (0.5 MOI) along with different doses of TMP269 (10 and 20 μM); cell samples were harvested at 24 hpi. As shown in Figure 8, TMP269 can significantly reduce the expression of M protein in N2a cells (Figure 8A), Vero cells (Figure 8B), and BHK-21 cells (Figure 8C) in a dose-dependent manner.

## Discussion

Rabies is a lethal zoonotic infectious disease with a mortality rate of almost 100% (Liu G. et al., 2021), and there are no effective treatment drugs. HATs and HDACs play critical roles in the regulation of gene expression through acetylating and deacetylating histones to regulate the chromatin structure, respectively. HDAC inhibitors (HDACi) have been extensively investigated in clinical trials in various types of cancers (Falkenberg and Johnstone, 2014). Recent studies suggest that the inhibition of HDAC activity can modulate viral replication (Zhou et al., 2015; Liu Z. et al., 2021). To investigate the role of TMP269 in RABV replication, we treated HEK-293T cells with TMP269 and infected with RABV. The results showed that TMP269 treatment significantly inhibited RABV replication at concentrations without significant cytotoxicity. Further study suggested that TMP269 can inhibit the autophagy process to decrease RABV replication.

HDAC inhibitors are a diverse group of small molecule drugs that increase the degree of acetylation of histones, involving in cell cycle, apoptosis, cell differentiation, autophagy, and anti-angiogenic process (Marks, 2010; Khan and La Thangue, 2012). The replication of several viruses has been reported to be modulated by HDACi. Moreover, the same HDACi has different effects on the replication of different viruses. For example, suberoylanilide hydroxamic acid (SAHA), which suppresses class I, II, and IV HDAC inhibitors, activates human immunodeficiency virus (HIV) replication in latently infected cells (Contreras et al., 2009) and coxsackievirus B3 (CVB3) replication in both cardiac myocytes and fibroblasts (Zhou et al., 2015). On the contrary, replication of hepatitis C virus (HCV) and human cytomegalovirus (HCMV) is suppressed by SAHA (Sato et al., 2013; Liu Z. et al., 2021). In our study, we found that TMP269 treatment significantly inhibited RABV replication in a dose-dependent manner at concentrations without significant cytotoxicity (Figure 1), and inhibition of HDAC activity with TMP269 significantly decreased RABV replication at an early stage in the viral life cycle (Figure 2). However, a further in-depth study is still needed. It remains to be revealed whether TMP269 has inhibitory effects through other mechanisms, such as the inhibition of RNA polymerase activity and/or activation of other host cell signaling pathway functions. Those findings demonstrated that TMP269 may provide a novel treatment strategy for rabies.

Innate immune response plays a vital role in preventing RABV infection (Katz et al., 2017). Host pattern recognition receptors (PRRs), such as the RIG-I-like receptor, NOD-like receptor, and Toll-like receptor, recognize pathogen-associated molecular patterns (PAMPs) of the virus to initiate the innate immune response (Akira et al., 2006). Contrary to our expectation, RNA sequencing showed that many innate immune-related pathways (Figure 5), such as the RIG-I-like receptor signaling pathway and Toll-like receptor signaling pathway, were repressed by treatment with TMP269, which demonstrated that the inhibition effect of TMP269 on RABV replication was independent of the innate immune response. This result is similar to the study by Liu Z. et al. (2021), in which SAHA suppressed the HCMV replication independent of the innate immune response. As we all know, excessive inflammatory response has been associated with RABV-induced diseases. In this study, we found that cytokines, including IL32, TNF, CCL2, CCL5, CCL20, CXCL3, CXCL8, and CXCL10, were downregulated by the treatment with TMP269, which demonstrated that TMP269 inhibits inflammation response caused by RABV at an early stage of infection. In addition, the underlying mechanism involved in TMP269-repressed innate immune response needs to be further explored.

Autophagy has been found to play critical roles in facilitating or inhibiting viral replication in multiple cellular signaling pathways, such as the MAPK signaling pathway and PI3K-AKT–mTOR signaling pathway (Campbell et al., 2018; Li et al., 2018). Many protein components are involved in the formation of autophagosomes, such as Beclin-1, Atg5, Atg12, Atg16L1, Atg7, and Atg3 (Levine and Kroemer, 2008). To investigate the mechanisms underlying the antiviral effect of TMP269 on RABV, we used bioinformatics to focus on the relative pathways or cellular factors. In this study, the DEGs in the TMP269 treatment group were involved in the MAPK signaling pathway, PI3K-AKT signaling pathway, and AMPK signaling pathway (Figure 5). In addition, We found that TMP269 treatment can inhibit the expression of ATG14, ATG10, ATG4C, ATG12, ATG5, ATG101, SQSTM1, BECN1, ATG7, ATG13, and ATG3 by RABV infection (Figure 7). Therefore, we wonder if the inhibition effect of TMP269 on RABV replication was dependent on autophagy. RABV infection has been reported to induce autophagy during viral replication in mouse neuroblastoma cell lines, and autophagy is beneficial to viral replication (Liu et al., 2017). In this study, we found that TMP269 treatment can decrease the expression of LC3-II in a dose-dependent manner (Figure 7B). These data demonstrated that TMP269 inhibited RABV replication by inhibiting the autophagy process; however, the underlying mechanism involved in TMP269-repressed autophagy process needs to be further explored.

## Conclusion

Our results proved that TMP269 can significantly inhibit RABV replication in a dose-dependent manner by inhibiting autophagy. Thus, we identified the effect of TMP269 during RABV infection, which may provide a novel treatment strategy for rabies.

## Data availability statement

The datasets presented in this study can be found in the NCBI repository under the accession numbers PRJNA1024637.

## Author contributions

JY: Writing – original draft, Writing – review & editing, Conceptualization, Data curation, Formal analysis, Investigation, Validation. SW: Conceptualization, Data curation, Formal analysis, Investigation, Methodology, Validation, Writing – original draft, Writing – review & editing. SR: Investigation, Writing – review & editing. ZL: Validation, Writing – review & editing. JG: Writing – review & editing, Formal analysis. YS: Resources, Supervision, Writing – review & editing. XY: Resources, Supervision, Validation, Writing – review & editing. XW: Conceptualization, Data curation, Formal analysis, Funding acquisition, Investigation, Methodology, Project administration, Resources, Supervision, Validation, Writing – original draft, Writing – review & editing.
